# New and Old Genes Associated with Primary and Established Responses to Paclitaxel Treatment in Ovarian Cancer Cell Lines

**DOI:** 10.3390/molecules23040891

**Published:** 2018-04-12

**Authors:** Monika Świerczewska, Andrzej Klejewski, Maciej Brązert, Dominika Kaźmierczak, Dariusz Iżycki, Michał Nowicki, Maciej Zabel, Radosław Januchowski

**Affiliations:** 1 Department of Histology and Embryology, Poznan University of Medical Sciences, Święcickiego 6 St., 61-781 Poznań, Poland; m_swierczewska@wp.pl (M.Ś.); dominika.ka.poznan@gmail.com (D.K.); mnowicki@ump.edu.pl (M.N.); mzab@ump.edu.pl (M.Z.); rjanuchowski@ump.edu.pl (R.J.); 2 Department of Nursing, Poznan University of Medical Sciences, Smoluchowskiego 11 St., 60-179 Poznan, Poland; 3 Department of Obstetrics and Women’s Diseases, Poznan University of Medical Sciences, Smoluchowskiego 11 St., 60-179 Poznan, Poland; 4 Division of Infertility and Reproductive Endocrinology, Department of Gynecology, Obstetrics and Gynecological Oncology, Poznan University of Medical Sciences, Polna 33 St., 60-535 Poznań, Poland; maciejbrazert@ump.edu.pl; 5 Department of Cancer Immunology, Poznan University of Medical Sciences, Garbary 15 St., 61-866 Poznań, Poland; dmizy@ump.edu.pl

**Keywords:** ovarian cancer, paclitaxel resistance, new genes

## Abstract

Development of drug resistance is the main reason for low chemotherapy effectiveness in treating ovarian cancer. Paclitaxel (PAC) is a chemotherapeutic drug used in the treatment of this cancer. We analysed the development of PAC resistance in two ovarian cancer cell lines. Exposure of drug-sensitive cell lines (A2780 and W1) to PAC was used to determine the primary response. An established response was determined in PAC-resistant sublines of the A2780 and W1 cell lines. qRT-PCR was performed to measure the expression levels of specific genes. We observed decreased expression of the *PCDH9*, *NSBP1, MCTP1* and *SEMA3A* genes in the PAC-resistant cell lines. Short-term exposure to PAC led to increased expression of the *MDR1* and *BCRP* genes in the A2780 and W1 cell lines. In the A2780 cell line, we also observed increased expression of the *C4orf18* gene and decreased expression of the *PCDH9* and *SEMA3A* genes after PAC treatment. In the W1 cell line, short-term treatment with PAC upregulated the expression of the *ALDH1A1* gene, a marker of Cancer stem cells (CSCs). Our results suggest that downregulation of the *PCDH9*, *NSBP1*, *MCTP1* and *SEMA3A* genes and upregulation of the *MDR1, BCRP, C4orf18* and *ALDH1A1* genes may be related to PAC resistance.

## 1. Introduction

Epithelial ovarian cancer (EOC) is one of the most aggressive tumours of all gynaecological malignancies and is the fifth leading cause of death from gynaecological malignancies. At the time of diagnosis, the majority of patients have advanced intraperitoneal metastatic disease [1]. Although most cases of EOC are chemosensitive at the beginning of chemotherapy, most patients develop drug resistance during treatment [2]. The first line of chemotherapy always includes taxane (paclitaxel) and platinum compounds [3]. Unfortunately, most of the patients with an initially good response to chemotherapy develop drug resistance and require further treatment with other cytotoxic agents. Recently a new anticancer therapy like immune check point inhibitors, use of CAR T cells or bispecific antibodies are extensively investigated [4].

Paclitaxel (PAC) is a chemotherapeutic agent originally isolated from *Taxus brevifolia*. It belongs to the family of antimitotic anticancer agents and blocks mitosis through stabilizing microtubules by binding to β-tubulin subunits. Consequently, PAC blocks cell division and leads to apoptotic cell death [5]. Clinically, the drug is used in the treatment of many cancers, including ovarian cancer [6], breast cancer [7] and lung cancer [8]. Unfortunately, many cancers develop PAC resistance. Some cancer cells express different β-tubulin isotypes with lower sensitivity to PAC action [9]. Others express mutated β-tubulin with lower PAC affinity [10]. However, the most important mechanism of PAC resistance is overexpression of drug transporters from the ABC family. Among these mechanisms, the most important role is played by expression of glycoprotein P (P-gp, ABCB1), encoded by the *MDR1* gene [11], although expression of the ABCB4 protein encoded by the *MDR3* gene seems to also be involved in this phenomenon [12]. Previously, we also described the increased expression of several collagens in PAC-resistant cell lines, suggesting their role in resistance to this drug [13].

However, in some cases, drug resistance is difficult to explain on the basis of the expression profile of known genes involved in this process, which indicates that new genes can also be involved in this phenomenon. Recently, using microarray data, we identified new genes that can also be associated with PAC resistance, such as *PCDH9*, *NSBP1*, *MCTP1, SEMA3A* [14] and *C4orf18*.

PCDH9 (protocadherin 9) is a member of the protocadherin protein family, which consists of approximately 80 members [15]. These proteins are calcium-dependent adhesion proteins implicated in neural cell–cell interactions. In contrast to classical cadherins, PCDHs appear to have more varied physiological functions [16]. PCDH9 has been reported to be a candidate tumour suppressor gene. Decreased expression of PCDH9 has been reported in glioma [17,18], gastric cancer [19], hepatocellular carcinoma [20], prostate cancer [21] and ovarian cancer [22], among other neoplasms. Downregulation of PCDH9 expression usually correlates with disease progression and shorter survival of patients [17,18,19,21].

Nucleosomal Binding Protein 1 (NSBP1) belongs to the high-mobility group nucleosome-binding protein (HMGN) family. These proteins modulate the structure and function of chromatin, which facilitates and enhances transcription, histone modifications, replication and DNA repair [23]. It has been reported that NSBP1 binds to nucleosomes via a nucleosomal binding domain (NBD), unfolds chromatin, and modulates gene transcription [24]. Increased expression of NSBP1 has been observed in many human cancers, including clear cell renal carcinoma (ccRC) [25], prostate cancer [26], gliomas [27] and meningiomas [28], suggesting its oncogenic role. In meningiomas, knockdown of NSBP1 resulted in P-gp downregulation and increased sensitivity to temozolomide [28]. It has also been reported that knockdown of NSBP1 increased the sensitivity of oesophageal squamous cell carcinoma (ESCC) cells towards cisplatin (CIS) and regulated *MDR1* gene expression [29].

Multiple C2 transmembrane domain-containing protein 1 (MCTP1) contains two transmembrane regions and three C2 domains with high Ca^2+^ activity [30]. The C2 domain is a Ca^2+^-binding motif prevalent in proteins involved in membrane trafficking/exchange processes that are important for vesicle formation, receptor trafficking, neurotransmitter release and cell migration [31]. Varied expression of MCTP1 has been observed in colorectal cancer specimens [32].

SEMA3A is a member of the semaphorin family, which comprises soluble and membrane bound proteins that play a role in neuronal development, organogenesis, angiogenesis and cancer progression [33]. SEMAs are classified into eight classes. Class 3 SEMAs (SEMA3) are the only secreted SEMAs in vertebrates. Several members of class 3 SEMAs, including SEMA3A, have been characterized as anti-angiogenic agents [34]. The SEMA3 class consists of seven soluble proteins of ~100 kDa (designated by the letters A–G), which are secreted by different cells, including neurons, epithelial cells and tumour cells. SEMA3s act in a paracrine fashion by binding to neuropilins via a highly conserved amino-terminal 500-amino acid region in the SEMA3 protein called the Sema domain [35]. SEMA3A is a putative tumour suppressor and is often downregulated in different types of cancer, including gastric cancer [36], ovarian cancer [37] and tongue cancer [38]. In gastric and ovarian cancer, downregulation of SEMA3A expression is correlated with disease progression and poor prognosis [36,37].

According to various databases expression of C4orf18 (FAM198B) was observed in nerves and epithelium during development however the detailed role of this protein was not described. Previously, we described its expression in CIS- and topotecan (TOP)-resistant ovarian cancer cell lines [39]. To our knowledge, its expression has not been described in the PubMed database by other authors.

Most of the research involving the development of resistance to cytotoxic drugs is conducted with pairs of drug-sensitive and drug-resistant cell lines that have been exposed to a drug for at least a few months. Knowledge about the response to cytotoxic drugs after first contact with the drugs at the beginning of treatment is poor. The goals of our study were as follows: (1) to investigate the expression level of new and old genes involved in PAC resistance in PAC-resistant ovarian cancer cell lines and (2) to analyse the expression of these genes during the first days of exposure to PAC.

## 2. Results

### 2.1. Gene Expression Analysis in PAC-Resistant Cell Lines

**Figure 1:**
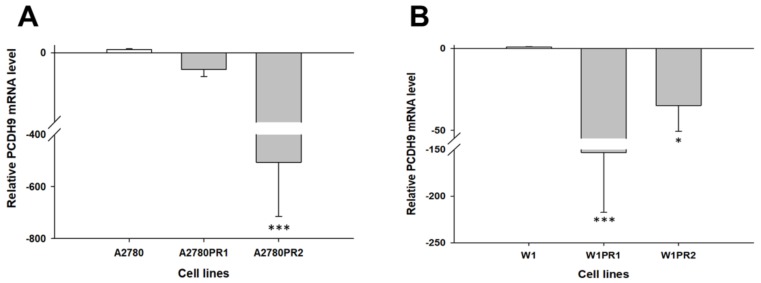
Expression analysis (Q-PCR) of the *PCDH9* gene in the A2780 (**A**) and W1 (**B**) PAC-resistant cell sublines. The figure presents the relative gene expression in the resistant cell lines (grey bars) with respect to that in the sensitive cell line (white bars), which was assigned a value of 1. The values were considered significant at * *p* < 0.05 and *** *p* < 0.001.

Our microarray data suggest that the *PCDH9*, *NSBP1*, *MCTP1*, *SEMA3A* [14] and *C4orf18* (not shown) genes may be involved in PAC resistance. The gene expression levels of *PCDH9*, *NSBP1*, *MCTP1*, *SEMA3A* and *C4orf18* were examined to determine whether the PAC resistance in our cell lines was associated with changed expression of these genes. We observed a statistically significant decrease in *PCDH9* transcript levels in the A2780PR2 cell line (*p* < 0.001) (Figure 1A) and in both W1 PAC-resistant cell lines (*p* < 0.001 in the W1PR1 cell line and *p* < 0.01 in the W1PR2 cell line) (Figure 1B).

**Figure 2:**
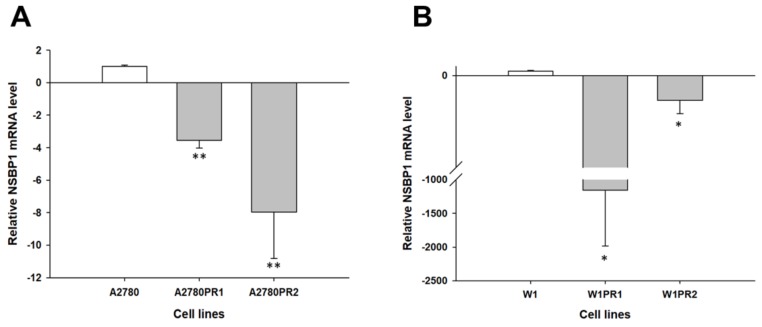
Expression analysis (Q-PCR) of the *NSBP1* gene in the A2780 (**A**) and W1 (**B**) PAC-resistant cell lines. The figure presents the relative gene expression in the resistant cell lines (grey bars) with respect to that in the sensitive cell line (white bars), which was assigned a value of 1. The values were considered significant at * *p* < 0.05 and ** *p* < 0.01.

Similar results were observed in the case of the *NSBP1* transcript. Decreased expression of *NSBP1* was seen in both A2780 PAC-resistant cell lines (*p* < 0.01) (Figure 2A) as well as in both W1 PAC-resistant cell lines (*p* < 0.05) (Figure 2B).

**Figure 3:**
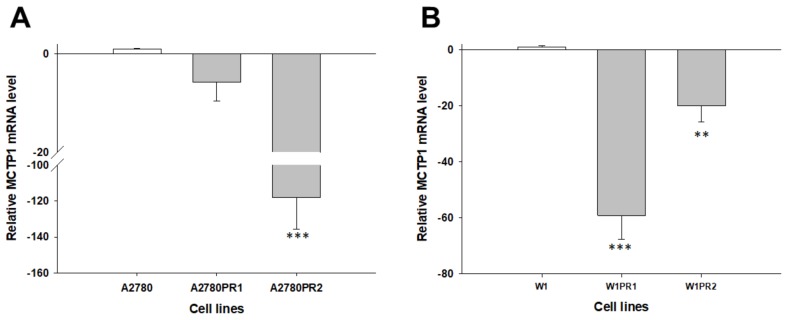
Expression analysis (Q-PCR) of the *MCTP1* gene in the A2780 (**A**) and W1 (**B**) PAC-resistant cell lines. The figure presents the relative gene expression in the resistant cell lines (grey bars) with respect to that of the sensitive cell line (white bars), which was assigned a value of 1. The values were considered significant at ** *p* < 0.01 and *** *p* < 0.001.

However, one should keep in mind that in the W1PR1 cell line, downregulation of the *NSBP1* transcript level was much lower than that in other PAC-resistant cell lines [approximately 1200-fold vs. 3-fold (W1PR2), 4-fold (A2780PR1) and 8-fold (A2780PR2)]. Downregulation of the *MCTP1* gene was observed in all PAC-resistant cell lines, although in the A2780PR1 cell line, downregulation of this gene was not statistically significant. In the A2780PR2 cell line, we observed approximately 120-fold downregulation of the *MCTP1* gene (*p* < 0.001) (Figure 3A). W1PR1 and W1PR2 were characterized by 60-fold (*p* < 0.001) and 20-fold (*p* < 0.01) downregulation of the *MCTP1* gene (Figure 3B).

**Figure 4:**
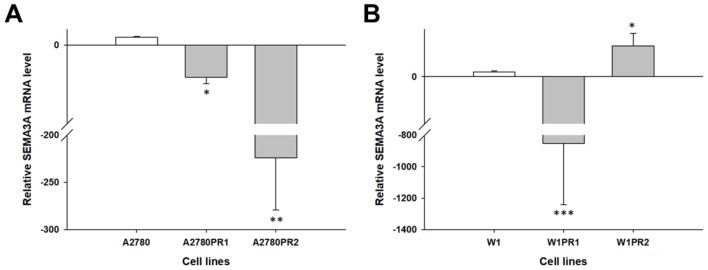
Expression analysis (Q-PCR) of the *SEMA3A* gene in the A2780 (**A**) and W1 (**B**) PAC-resistant cell lines. The figure presents the relative gene expression in the resistant cell lines (grey bars) with respect to that of the sensitive cell line (white bars), which was assigned a value of 1. The values were considered significant at * *p* < 0.05, ** *p* < 0.01 and *** *p* < 0.001.

A different pattern of *SEMA3A* gene expression was observed in PAC-resistant cell lines. A 4-fold decrease in the *SEMA3A* transcript level was observed in the A2780PR1 cell line (*p* < 0.05) vs. 223-fold in the A2780PR2 cell line (*p* < 0.01) (Figure 4A). Even larger differences were observed in the W1 PAC-resistant cell lines. In the W1PR1 cell line, we observed very high downregulation of the *SEMA3A* transcript level—approximately 850-fold (*p* < 0.001) (Figure 4B). In contrast, in the W1PR2 cell line, we observed a 6-fold increase in the *SEMA3A* transcript level (*p* < 0.05) (Figure 4A).

### 2.2. Early Response to PAC Treatment in Ovarian Cancer Cell Lines

The second part of our study focused on the early response to PAC treatment. In these experiments, drug-sensitive cell lines A2780 and W1 were treated with low concentrations of PAC (20 ng/mL and 25 ng/mL for A2780) and (15 ng/mL and 20 ng/mL for W1) for 24, 48 and 72 h. Then, changes in gene expression were investigated.

**Figure 5:**
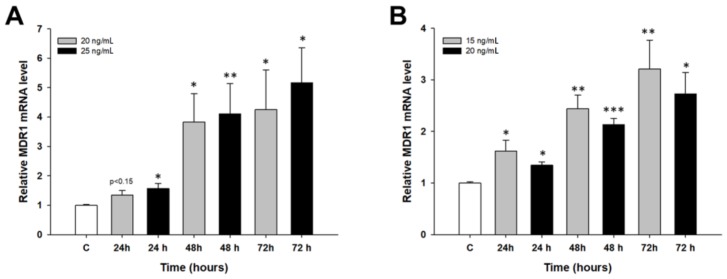
Expression analysis of the *MDR1* gene in the A2780 (**A**) and W1 (**B**) cell lines. The figure presents the relative gene expression in the PAC-treated cells (grey and black bars) with respect to that in the untreated control (white bars), which was assigned a value of 1. The values were considered significant at * *p* < 0.05, ** *p* < 0.01 and *** *p* < 0.001.

We were interested in whether *MDR1* and *BCRP* genes encoding the most important drug transporters can also be expressed during the first days of PAC treatment. In the A2780 cell line, we observed a dose- and time-dependent increase in the *MDR1* transcript level (*p* < 0.05 or *p* < 0.01) (Figure 5A). In the W1 cell line, we also observed a time-dependent increase in the *MDR1* transcript level after PAC treatment, although the increase was always higher at the 15 ng/mL concentration than at the 20 ng/mL concentration (*p* < 0.05, *p* < 0.01 or *p* < 0.001) (Figure 5B).

**Figure 6:**
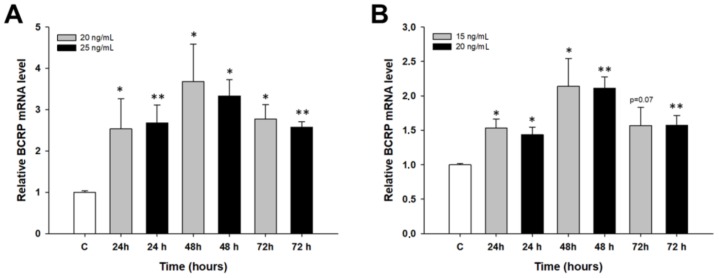
Expression analysis of the *BCRP* gene in the A2780 (**A**) and W1 (**B**) cell lines. The figure presents the relative gene expression in the PAC-treated cells (grey and black bars) with respect to that in the untreated control (white bars), which was assigned a value of 1. The values were considered significant at * *p* < 0.05 and ** *p* < 0.01.

In the A2780 cell line, a statistically significant increase in the *BCRP* transcript level was observed at all time points and concentrations (*p* < 0.05 or *p* < 0.01) (Figure 6A). Similar results were observed in the W1 cell line (*p* < 0.05 or *p* < 0.01), with the exception of PAC 15 ng/mL after 72 h, for which the increase was close to significant (*p* = 0.07) (Figure 6B).

**Figure 7:**
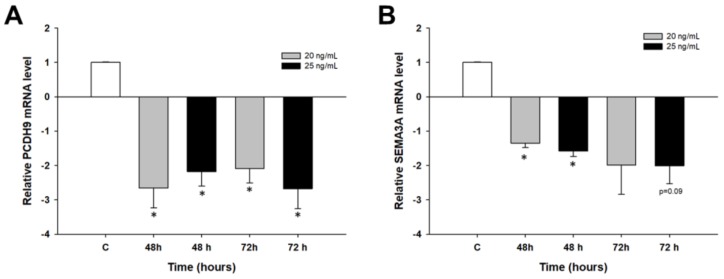
Expression analysis of the *PCDH9* gene (**A**) and *SEMA3A* gene (**B**) in the A2780 cell line. The figure presents the relative gene expression in the PAC-treated cells (grey and black bars) with respect to that of the untreated control (white bars), which was assigned a value of 1. The values were considered significant at * *p* < 0.05.

In both cell lines, the highest transcript level was observed after 48 h of treatment. The expression levels of *PCDH9* and *SEMA3A* genes were investigated to determine if PAC could induce changes in the expression of these genes during the first days of treatment. We did not observe any changes in the expression of these genes in the W1 cell line (not shown). In the A2780 cell line, we observed statistically significant downregulation of the *PCDH9* gene after 48 and 72 h of PAC treatment (*p* < 0.05) (Figure 7A). Downregulation of the *SEMA3A* transcript level was statistically significant (*p* < 0.05) only after 48 h of treatment and was close to significant (*p* = 0.09) after 72 h of treatment at a PAC concentration of 25 ng/mL (Figure 7B).

**Figure 8:**
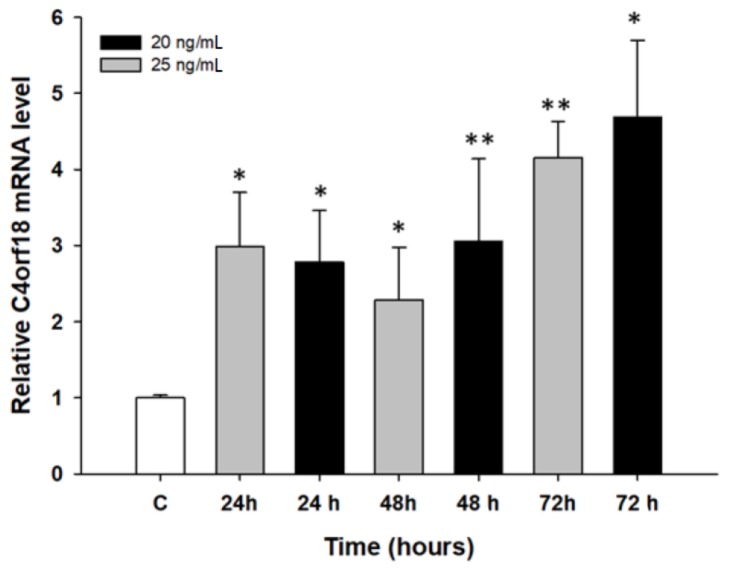
Expression analysis of the *C4orf18* gene in the A2780 cell line. The figure presents the relative gene expression in the PAC-treated cells (grey and black bars) with respect to that of the untreated control (white bars), which was assigned a value of 1. The values were considered significant at * *p* < 0.05 and ** *p* < 0.01.

Increased expression of the *C4orf18* gene after PAC treatment was observed in only the A2780 cell line. At all time and concentration points, we observed statistically significant expression, with the highest *C4orf18* transcript levels after 72 h of treatment (*p* < 0.05 and *p* < 0.01) (Figure 8).

**Figure 9:**
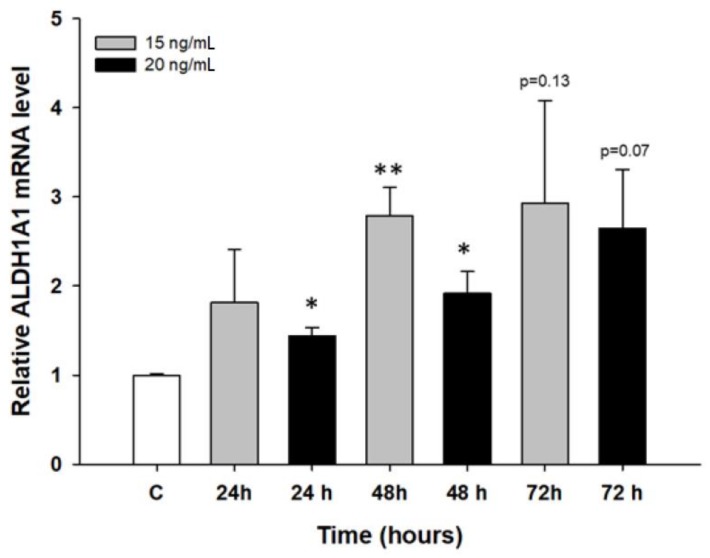
Expression analysis of the *ALDH1A1* gene in the W1 cell line. The figure presents the relative gene expression in the PAC-treated cells (grey and black bars) with respect to that of the untreated control (white bars), which was assigned a value of 1. The values were considered significant at * *p* < 0.05 and ** *p* < 0.01.

ALDH1A1 is the most popular marker of cancer stem cells (CSCs), and its expression was previously observed by us in PAC- and TOP-resistant cell lines [40]. Therefore, we were interested in whether the expression of this gene can increase after short-term PAC treatment. We did not observe any changes in *ALDH1A1* transcript in the A2780 cell line (not shown). In contrast, we observed an increase in *ALDH1A1* mRNA after PAC treatment in W1 cell line. The increase was statistically significant after 24 and 48 h at a PAC concentration of 20 ng/mL and after 48 h at a concentration of 15 ng/mL (*p* < 0.05 or *p* < 0.01). After 72 h, the increase was close to significant (*p* = 0.13 for 15 ng/mL and *p* = 0.07 for 20 ng/mL) (Figure 9).

## 3. Discussion

Ovarian cancer is a good model for studying drug resistance development. Although in most cases ovarian cancer responds well to chemotherapy at the beginning of treatment, development of drug resistance during chemotherapy leads to high mortality among ovarian cancer patients [2]. In this study, we investigated the response to PAC treatment and the development of PAC resistance in ovarian cancer cell lines. PAC is used in combination with platinum compounds (CIS or carboplatin) as a first line of ovarian cancer chemotherapy [3]. From published data, it is known that resistance to PAC is mainly associated with expression of P-gp [11] and/or expression of different β-tubulin isotypes [9] or mutation of β-tubulin genes [10]. Previously, we also described increased expression of *MDR1* and *MDR3* genes [12] and several collagen genes [13] in investigated PAC-resistant cell lines. Here, we describe the expression of five new genes that may be involved in PAC resistance.

We observed statistically significant downregulation of the *PCDH9* gene in three of four PAC-resistant cell lines. This finding suggests that decreased expression of this gene may be related to PAC resistance in ovarian cancer. While searching published data, we did not find any report concerning the role of PCDH9 in drug resistance. However, there are many reports describing *PCDH9* as a tumour suppressor gene in many cancers. Downregulation of PCDH9 expression in glioma has been reported by Wang. Expression of PCDH9 was downregulated in glioma in comparison to normal brain tissue, and loss of PCDH9 expression was associated with a higher histological grade and shorter survival time of patients [17,18]. Reduced expression of PCDH9 was also observed during the progression of gastric cancer. Chen et al. observed a reduction in the PCDH9 level from normal gastric mucosa to primary tumour and from nodal metastasis to liver metastasis. PCDH9 expression is inversely correlated with tumour size, tumour differentiation, tumour invasion, lymph node metastasis, histological grade and patient survival [19]. In hepatocellular carcinoma, downregulation of PCDH9 expression is correlated with portal vein invasion, increased tumour cell migration and increased EMT [20]. Downregulation of PDH9 expression has also been reported in prostate cancer and is correlated with disease progression, elevated levels of prostate-specific antigen and a high clinical stage of prostate tumours [21]. Decreased level of PCDH9 transcript was also observed in ovarian cancer tumours in comparison to normal ovarian epithelium [22]. Restoration of PCDH9 expression leads to a significant reduction in cell viability [18] proliferation [21], migration [20,21], and invasion [21]; blocks EMT [20]; and leads to increased apoptosis [18]. An in vivo study showed markedly reduced tumour growth with the expression of PCDH9 in comparison to the control [21]. All these results indicate that decreased/loss of PCDH9 expression correlates with different features of cancer disease progression. None of these studies addressed the effect of the loss of PCDH9 expression on cytotoxic drug sensitivity. However, because the expression of PCDH9 is decreased in poorly differentiated cancer tissues and metastases, and metastases are usually more resistant to chemotherapy, and PCDH9 expression was decreased in PAC-resistant cell lines, it is possible that decreased expression of PCDH9 in different cancers and cancer cell lines also leads to chemotherapy resistance. According to model presented by Zhu et al. [20] PCDH9 inhibit the activity of Akt and ERK kinases. Wang et al. observed that expression of PCDH9 arrest cells at the G0/G1 phase and reduce the number of cells in S phase by inhibiting cyclin D1 and Bcl-2 expression [17]. In result downregulation of PCDH9 observed in our cell lines can lead to higher kinases activity, resulting in higher signal transduction, higher drug resistance and cell cycle genes expression leading to increased cells proliferation and tumor progression. This possibility, however, requires additional study.

Downregulation of *NSBP1* expression was observed in all PAC-resistant cell lines, although very high downregulation (over 1000-fold) was observed in only the W1PR1 cell line. Downregulation in all PAC-resistant cell lines suggests that *NSBP1* may indeed be responsible for PAC resistance. Loss of NSBP1 expression was also associated with higher gemcitabine resistance in prostate cancer [41]. In contrast to these and our results, it was previously observed that inhibition of *NSBP1* expression sensitized meningioma cell lines to temozolomide and decreases the expression of P-gp [28]. Knockdown of NSBP1 also resulted in significantly decreased expression of P-gp in ESCC cells, whereas overexpression of NSBP1 significantly increased the expression of P-gp [30]. NSBP1 seems to also be associated with CIS resistance in ESCC because knockdown of NSBP1 markedly decreased CIS sensitivity and overexpression of NSBP1 markedly increased CIS resistance [30]. In summary, we observed two opposite effects. Low NSBP1 expression is associated with higher resistance to PAC in ovarian cancer and higher resistance to gemcitabine in prostate cancer [41]. In contrast, in meningioma and ESCC, a high expression level of NSBP1 is associated with resistance to temozolomide or CIS, respectively [28,29]. In meningioma and ESCC, NSBP1 seems to be a positive regulator of the *MDR1* gene encoding P-gp [28,29]. In contrast to these studies, we observed downregulation of *NSBP1* in all PAC-resistant cell lines, although expression of the *MDR1* gene was upregulated in investigated cell lines, as we reported previously [12]. Thus, the role of NSBP1 in cancer drug resistance seems to be cancer type specific. In a clinical study, increased expression of NSBP1 was associated with histological grade in meningioma [28] and ccRCC [25] and was upregulated in ESCC [29] bladder cancer [42] and NSCLC [43]. Knockdown of NSBP1 was associated with reduced cell proliferation and growth in vivo in prostate cancer [44], inhibition of invasion in ccRCC [25], inhibition of EMT in bladder cancer [42] and reduced proliferation and invasion in NSCLC [43]. Molecularly downregulation of NSBP1 resulted in decreased growth of cells and G2/M cell cycle arrest in prostate cancer [44], downregulation of Bcl-2 in ESCC [29] and downregulation of B1 cyclin in ESCC [29] and NSCLC [43]. Thus, expression of NSBP1 plays an important role in the progression of many cancers. The relationship between NSBP1 expression and cancer drug resistance development requires further investigation.

In three of four PAC-resistant cell lines, we observed statistically significant decreased expression of the *MCTP1* gene. These results suggest that downregulation of this gene is specific to resistance to PAC. MCTP1 is poorly described in the literature. It belongs to proteins containing a C2 Ca^++^-binding motif involved in membrane trafficking/exchange processes, such as cell migration, receptor trafficking and vesicle formation [30,31]. Previously, we described the downregulation of this gene in CIS- and TOP-resistant ovarian cancer cell lines [39]. Here, we observed downregulation in PAC-resistant cell lines. Thus, downregulation of MCTP1 expression can be an unspecific marker of drug resistance development. So far, expression of MCTP1 has been investigated in only colorectal cancer with differential expression but without any clinical correlation [32]. Thus, the significance of MCTP1 expression in cancer progression and drug resistance requires further investigation.

Downregulation of the *SEMA3A* gene was observed in three of four PAC-resistant cell lines, suggesting the importance of *SEMA3A* gene downregulation in PAC resistance development. To the best of our knowledge, changes in *SEMA3A* gene expression have not been correlated with cancer drug resistance so far. However, downregulation of SEMA3A has been observed in many cancers and correlates with clinicopathological features. Decreased expression of SEMA3A was observed in gastric tumour tissue in comparison to a control. Furthermore, low expression of SEMA3A was significantly correlated with poor differentiation, vascular invasion, depth of invasion, lymph node and distant metastasis and advanced TNM stage. Low SEMA3A expression was also significantly correlated with poor patient prognosis. Overexpression of SEMA3A in the gastric cancer cell line SGC-7901 inhibited cell proliferation and migration in vitro [36]. Significant downregulation of SEMA3A expression was also observed in epithelial ovarian cancer in comparison to normal epithelium. A low level of SEMA3A was correlated with higher clinical advancement (FIGO), poor histological grade, lymph node and distinct metastasis and poor patient prognosis [37]. SEMA3A expression was also significantly downregulated in tongue cancer and correlated with nodal metastasis. Lower SEMA3A expression strongly predicted shorter patient survival [38]. Thus, downregulation of SEMA3A expression was correlated with metastasis [36,37,38] and poor tumour tissue differentiation [36,37]. Poorly differentiated and tumours and metastases are usually more resistant to chemotherapy. It seems possible that lower SEMA3A expression in poorly differentiated tumours and metastatic tumour tissue may be associated with drug resistance; this possibility, however, requires further investigation. Mechanistically reduced SEMA3A expression can result in higher proteins phosphorylation and enhanced signal transduction. It has been observed that overexpression of SEMA3A in the tongue squamous cell carcinoma cell line SSC-9 result in suppressed tumor growth in mice and inhibition of VEGFR2, Src and FAK phosphorylation [45]. The other question is regarding the very large difference in SEMA3A expression between W1 PAC-resistant sublines W1PR1 and W1PR2. Both cell lines were developed in identical conditions from the same parental cell line. In the W1PR1 subline, we observed a strong decrease in the *SEMA3A* transcript level (approximately 900-fold). In contrast, in the W1PR2 cell line, we observed an increase in the *SEMA3A* transcript. Both cell lines are very resistant to PAC, and both express the *MDR1* gene, encoding P-gp, at very high levels [12]. Similar differences between two cell lines resistant to the same drug were observed by our group previously. Although two cell lines developed under the same conditions from the same parental cell line, they presented different profiles of gene expression and different drug cross-resistance [11]. This finding indicates the complexities of cancer development. From hundreds of genes that may be important in drug resistance, cells choose some. The main mechanisms of resistance are usually the same (P-gp expression in the case of PAC resistance), and less-important (complementary) mechanisms of drug resistance can be different. In the second part of our study, we wanted to determine if the same genes are expressed after the first exposure to PAC treatment at the beginning of PAC resistance development. The most important gene in PAC resistance is *MDR1*, encoding P-gp [46]. We also previously described the expression of this gene in PAC-resistant ovarian cancer cell lines [11,12]. Here, we observed that expression of the *MDR1* gene started after 24 h of PAC exposure and increased in a time-dependent manner in the A2780 and W1 cell lines. Upregulation of the *MDR1* gene after the first contact with PAC confirms the significance of this gene expression in PAC resistance. The second most important drug transporter is BCRP. Its expression is related to TOP resistance in many cancers, including ovarian cancer [47], and was described by our group in TOP-resistant ovarian cancer cell lines [11,48] and after short-term exposure to TOP treatment [49]. Here, we observed increased expression of *BCRP* after short-term exposure to PAC, with a maximum level after 48 h of treatment. To our knowledge, PAC is not a substrate of the BCRP drug transporter [46], and we did not observe increased expression of BCRP in PAC-resistant cell lines [11,48]. On the other hand, drug transporters from the ABC family are characterized by low substrate specificity [46]. It is possible that after the first contact with PAC, cells increase the expression of both transporters (BCRP and P-gp) to increase their chance to survive. Then, when the expression of P-gp increases to a level that protects the cells, the expression of the less-effective BCRP declines, as we observed. Next, we were interested in determining if the expression of “new” genes can also be changed after short-term exposure to PAC. We did not observe any changes in *NSBP1* gene expression after short-term PAC exposure, suggesting that *NSBP1* is not important at the beginning of drug resistance development. We observed statistically significant downregulation of the *PCDH9* gene after 48 and 72 h of PAC treatment in the A2780 cell line. No changes were observed in the W1 cell line. Downregulation of *SEMA3A* gene expression was also observed in only the A2780 cell line after 48 and 72 h of treatment, with no changes observed in the W1 cell line. This finding suggests that downregulation of *PCDH9* and *SEMA3A* expression after short-term PAC exposure is important only in the A2780 cell line. Downregulation after short-term exposure in PAC-resistant cell lines indicates the significance of downregulation of these genes in PAC resistance development. *C4orf18* is a poorly described gene. Previously, we observed increased expression of this gene in CIS- and TOP-resistant sublines of the A2780 cell line. Expression of this gene was also observed after short-term CIS or TOP exposure [39]. Here, we observed increased expression after short-term PAC exposure, but in only the A2780 cell line. However, we did not observe any changes in *C4orf18* gene expression in PAC-resistant cell lines (not shown). Therefore, we can conclude that expression of the *C4orf18* gene is specific to only the A2780 cell line and is important at the beginning of MDR development. Furthermore, *C4orf18* expression may be drug type specific. Development of drug resistance can be explained by different models. One of the most popular is the cancer stem cell model [50]. According to the CSC model of drug resistance development, a small population of cells is resistant to chemotherapy, and after exposure to drugs, these cells survive. The most popular marker of CSCs is expression of aldehyde dehydrogenase 1A1 (ALDH1A1). According to this model, increased expression of *ALDH1A1* after PAC exposure can result not only from increased expression but also from changes in the proportion between CSCs and other cell populations. The second alternative is even more probable because previously we observed a very high population of ALDH+ cells in a W1PR1 cell line resistant to PAC in comparison to a PAC-sensitive W1 cell line [40]. An ALDH+ population was also observed in the A2780PR1 cell line (under review). Thus, increased drug resistance can result from an increased number of CSCs in PAC-resistant cell lines. Increased expression of *ALDH1A1* can also result from downregulation of *PCDH9*. It has been observed that overexpression of PCDH9 downregulates the expression of ALDH1A1 in prostate cancer cell lines [21].

Here we discussed the expression of six genes that can be involved in PAC-resistance development in ovarian cancer cell lines. The significance of these genes in primary and established PAC resistance can be clearer in the correlation with another genes expression. Thus, in the future we are going to perform the RNA microarray analysis to determine changes in gene expression pattern in primary and established response to PAC-treatment. Because our study is a cell line study it has some limitations. These limitations can be resolved by animal study [51,52]. In next step we are going to perform in vivo experiments using mouse to determine the significance of investigated genes in drug resistance. Animal study using cell lines with overexpression or knock down of investigated genes should offer important information about the significance of their expression in drug resistance. In standard cell culture condition cells develop mainly cell specific mechanism of drug resistance. In animal study cells form tumor tissue like structure. In such structure another mechanism of drug resistance like limited drug diffusion or cell adhesion mediated drug resistance (CAM-DR) can be important [53]. Thus, in vivo experiments should increase our knowledge about the role of investigated genes in drug resistance.

During the last decade a big progress in cancer immunotherapy has been made [4]. Immunotherapy can be also effective in case of drug resistance cancers. In immunomodulation strategy antibody can restore and increase response against chemotherapy resistant cancers [54]. The chimeric antigen receptor T (CAR-T) cells can eliminate tumor cells by binding of tumor-associated antigens [55].

Our drug resistant cell lines can be also used as a model to test a new anticancer compound in in vitro and in vivo study using patient derived xenografts [52]. NT1014 is a new chemical compound that was used in study using ovarian cancer cell lines. It inhibited cell proliferation and lead to apoptosis in vitro and in vivo [56]. It would be interesting to test if our PAC-resistant cell lines are sensitive or resistant to this agent. In conclusion, different anticancer therapies should be use together to increase effectives and make cancer a treatable disease in most cases.

## 4. Materials and Methods

### 4.1. Reagents

PAC, RPMI-1640 and MEM media, foetal bovine serum, antibiotic-antimycotic solution, and L-glutamine were purchased from Sigma (St. Louis, MO, USA).

### 4.2. Cell Lines and Cell Culture

In our study, we used two ovarian cancer cell lines: the established ovarian cancer cell line A2780 and the primary ovarian cancer cell line W1. The human ovarian carcinoma A2780 cell line was purchased from ATCC (Manassas, VA, USA). A2780 sublines that were resistant to PAC [A2780PR1 and A2780PR2 (A2780 paclitaxel resistant)] were generated by exposing A2780 cells to PAC at incrementally increasing concentrations. The human primary ovarian cancer cell line W1 was established using ovarian cancer tissue obtained from an untreated patient. The W1 sublines resistant to PAC [W1PR1 and W1PR2 (W1 paclitaxel resistant)] were obtained by exposing W1 cells to PAC at incrementally increasing concentrations. The final concentrations used for selecting the resistant cells was 1100 ng/mL of PAC and was two-fold higher than the plasma concentrations of PAC two hours after intravenous administration. The increase in resistance according to parental drug-sensitive cell lines were as follows: 146-fold for A2780PR1 vs. A2780; 1202-fold for A2780PR2 vs. A2780; 743-fold for W1PR1 vs. W1 and 967-fold for W1PR2 vs. W1 as described previously [12,48]. All the cell lines were maintained as monolayers in complete medium [MEM (A2780) and RPMI-1640 medium (W1) supplemented with 10% (*v*/*v*) foetal bovine serum, 2 pM l-glutamine, penicillin (100 units/mL), streptomycin (100 units/mL) and amphotericin B (25 μg/mL)] at 37 °C in a 5% CO_2_ atmosphere.

### 4.3. Incubation of Cells with PAC

In time course experiments, the A2780 and W1 cell lines were treated with PAC at concentrations of 20 ng/mL and 25 ng/mL for A2780 or 15 ng/mL and 20 ng/mL for W1. The starting cell concentration was 0.4 × 10^6^ (A2780 and W1) in 1 mL of medium per well of a 6-well plate. Cell count and viability were determined before the cells were used in the different assays. Viability was determined by the trypan blue exclusion criteria. Cells were harvested and used for RNA isolation 24, 48 and 72 h after treatment.

### 4.4. Examination of Gene Expression by Q-PCR

Changes in *PCDH9*, *MCTP1*, *NSBP1*, *SEMA3A, C4orf18*, *ALDH1A1, MDR1* and *BCRP* gene expression in A2780 and W1 cell lines as well as in the PAC-resistant cell lines were examined. RNA was isolated using a Gene Matrix Universal RNA Purification Kit (EURx, Ltd., Gdańsk, Poland) as described by the manufacturer’s protocol. Reverse transcription was performed using M-MLV reverse transcriptase (Invitrogen by Thermo Fisher, Waltham, MA, USA) as described in the manufacturer’s protocol using a thermal cycler (Veriti 96-well Thermal Cycler, Applied Biosystems, 850 Lincoln Centre Drive, Foster City, CA, USA); 2 μg of RNA was used for cDNA synthesis. Real-time PCR was performed using a 7900HT Fast Real-Time PCR System (Applied Biosystems, 850 Lincoln Centre Drive, Foster City, CA, USA), Maxima SYBR Green/ROX qPCR Master Mix (Thermo Fisher Scientific, Waltham, MA, USA) and sequence-specific primers, as indicated in Table 1. Glyceraldehyde-3-phosphate dehydrogenase (*GADPH*), β-actin, hypoxanthine-guanine phosphoribosyltransferase 1 (*HRPT1*) and beta-2-microglobulin (*β2M*) served as the normalizing genes (geometric mean) against which changes in the expression of examined genes were compared. Gene expression was analysed using the relative quantification (RQ) method. RQ estimates the differences in the level of gene expression against a calibrator (RQ of the calibrator = 1). The drug-sensitive cell lines (A2780 and W1) were used as the calibrators. The analysis was conducted by employing the standard formula: RQ = [sample (drug-resistant line) calibrator (drug-sensitive line)]. The graphs were made using SigmaPlot (Systat Software GmbH Schimmelbuschstrasse 25 D-40699, Erkrath, Germany).

For amplification, 12.5 μL of Maxima SYBR Green/ROX qPCR Master Mix (Fermentas by Thermo Fisher, Waltham, MA, USA), 1 μL of each primer (Oligo, Warsaw, Poland, Table 1), 9.5 μL of water, and 1 μL of cDNA solution were mixed together.

One RNA sample from each preparation was processed without the RT-reaction to serve as a negative control in the subsequent PCR. Sample amplification included a hot start (95 °C, 15 min) followed by 45 cycles of denaturation at 95 °C for 15 s, annealing at 60 °C for 30 s, and extension at 72 °C for 30 s. After amplification, melting curve analysis was performed to analyse the melting temperature of the product. The amplification products were also resolved by 3% agarose gel electrophoresis and visualized by ethidium bromide staining.

### 4.5. Statistical Analysis

The statistical analysis was performed using Excel software (Microsoft, Redmont, WA, USA). The statistical significance of the differences was determined by applying Student’s *t*-test. 

## 5. Conclusions

In summary, our results show the changes in the expression of new genes in response to PAC treatment in ovarian cancer cell lines. The decreased expression of *PCDH9*, *NSBP1*, *MCTP1* and *SEMA3A* genes suggests that downregulation of these genes is important in PAC resistance. Upregulation of the *BCRP* and *C4orf18* genes was characteristic of only the primary response to PAC. The significance of these genes in the resistance to PAC has not been described previously and should be further investigated. We also confirmed the significance of the *MDR1* gene in the PAC response. Upregulation of the *ALDH1A1* gene suggests the role of CSCs in PAC resistance development in the W1 cell line.

## Figures and Tables

**Table 1:** Oligonucleotide sequences used for Q-PCR analysis.

Transcript	Sequence (5′–3′ Direction)		ENST Number	Product Size
MCTP1	F	AGAACCTCAACCCTGTGTGG	00000312216	123 bp
	R	AGGCTGAGCCCATAAAGTCA		
PCDH9	F	CTCTCCGGACAAGAGGACTG	00000544246	125 bp
	R	AGTGACCCAAAACCAAGCAC		
NSBP1	F	TGTGCCAGTTACACCAGAGG	00000358130	124 bp
	R	TTGCTTGGTTTCAGCAACTG		
C4orf18	F	GAGTACCCAAGCCTGAATCG	00000393807	137 bp
	R	ATCTTCCTTGCGAGGTCTGA		
SEMA3A	F	TGTTGGAGCAAAGGATCACA	00000265362	109 bp
	R	AGCCCACTTGCATTCATCTC		
ALDH1A1	F	GTTGTCAAACCAGCAGAGCA	00000165092	115 bp
	R	CTGTAGGCCCATAACCAGGA		
MDR1	F	TGACAGCTACAGCACGGAAG	00000265724	131 bp
	R	TCTTCACCTCCAGGCTCAGT		
BCRP	F	TTCGGCTTGCAACAACTATG	00000237612	128 bp
	R	TCCAGACACACCACGGATAA		
GAPDH	F	GAAGGTGAAGGTCGGAGTCA	00000229239	199 bp
	R	GACAAGCTTCCCGTTCTCAG		
β-actin	F	TCTGGCACCACACCTTCTAC	00000331789	169 bp
	R	GATAGCACAGCCTGGATAGC		
HPRT1	F	CTGAGGATTTGGAAAGGGTG	00000298556	156 bp
	R	AATCCAGCAGGTCAGCAAAG		
Β2M	F	CGCTACTCTCTCTTTCTGGC	00000558401	133 bp
	R	ATGTCGGATGGATGAAACCC		
